# Supplementary material for: Simulations of Tubulin Sheet Polymers as Possible Structural Intermediates in Microtubule Assembly
Source: PLoS One. 2009 Oct 2;4(10):e7291. doi: 10.1371/journal.pone.0007291 (PMC2752796; doi:10.1371/journal.pone.0007291)
Supplement: Supporting Text S1 — A. Rate constants in the model. B. Calculation of the entropic contribution. C. Physical origins of the temperature dependence of the free energy terms. (0.32 MB DOC) [file pone.0007291.s001.doc]

## Supporting Text S1

**A. Rate constants in the model:** As discussed in the main text, three types of basic reactions are considered in our model (Fig. 2a). The following formula give the corresponding reaction rates satisfying detailed balance:

1) Longitudinal On/Off: The reaction rate constants for dimer addtion/dissociation from the plus end of a protofilament are given by

$$k_{L}^{N\to N+1}=k_{L}^{1\to2}$$

$$k_{-L}^{N+1\to N}=k_{L}^{N\to N+1}\exp\left(\left(\Delta G_{long}+i\Delta G_{lat}+\Delta G_{Entropy}(N\to N+1)\right)/k_{B}T\right)$$

where $k_{-L}$ is the disassociating rate, related to the associating rate $k_{L}$ by the effective binding free energy. We assume that the association rate is independent of the protofilament length. $\Delta G_{long}$is the longitudinal bond binding energy, $\Delta G_{lat}$is the binding energy for a lateral bond, which can be either *tube* bond or sheet bond. The corresponding values are $\Delta G_{Tu}$ and$\Delta G_{Su}$. The number *i* refers to the number of lateral bonds formed during the association process, which ranges from 0 to 2. $\Delta G_{Entropy}$ accounts for lose of translational and rotational entropy during the association process, which is defined as positive. $\Delta G_{Entropy}(N\to N+1)$represents the entropic portion of the energy corresponding to adding one dimer onto the cluster with N dimers. For small N, $\Delta G_{Entropy}(N\to N+1)$also depends on the cluster shape. Its calculation is discussed below. The term $k_{B}$is the Boltzmann constant, and $T$ is the temperature in Kelvin. The minus end longitudinal reactions are the same as plus end reactions, except for a constant factor$\delta$ ~ 0.3 [1]

2) Lateral On/Off: The rate constants for one dimer lateral association or disassociation from the cluster are

$$\begin{matrix} k_{-Tu}=k_{T}\exp\left(\Delta G_{Tu}/k_{B}T+\Delta G_{Entropy}(1\to2)/k_{B}T\right), \\ k_{-Sh}=k_{S}\exp\left(\Delta G_{Sh}/k_{B}T+\Delta G_{Entropy}(1\to2)/k_{B}T\right), \end{matrix}$$

Where subscript *Tu* stands for *tube* binding, and *Sh* stands for sheet binding. Lateral association rates *k_Tu_* and *k_Sh_* are determined by comparing existing model parameters and proposed mechanism. *k_-Tu_* and *k_-Sh_* are disassociation rates. Free energies are defined in the same way as for the longitudinal reaction.

3) Switch of lateral bonds: The conversion rate constants between *sheet* bond and *tube* bond are given by

$k_{Tu\to Sh}^{n}=k^{0}\exp\left(n\left(\Delta G_{Tu}+\varepsilon\Delta G_{TuTu}-\Delta G^{+}\right)/k_{B}T\right),$.

$$k_{Sh\to Tu}^{n}=k^{0}\exp\left(n\left(\Delta G_{Sh}+\zeta\Delta G_{ShSh}-\Delta G^{+}\right)/k_{B}T\right)$$

where the activation energy, $\Delta G^{+}>\Delta G_{Tu},\Delta G_{Sh}$, is the energy barrier between two bond types, $k^{0}$is a constant, $\Delta G_{ShSh}$and $\Delta G_{TuTu}$ are the allosteric terms in Scheme 1 and Shceme 2 (see supporting text C and also in main text), respectively. The parameters $\varepsilon$ and $\zeta$ can assume values 0, 1, or 2, depending on the lateral bond types of neighbor filament pairs. For instance, $\zeta=0$if both neighbor filament pairs have tube bonds. The switching rates decrease quickly with increasing number of lateral bonds.

**B. Calculation of the entropic contribution:** Erickson discussed the necessity of treating different free energy contributions, especially the translational and rotational entropy, separately [2]. He discussed the situation adding one tubulin dimer to a large growing microtubule. In our case the system starts with dimers, and form larger and larger clusters. Therefore, we will need to generalize the procedure of Erickson, as discussed below.

The entropic term appears in both longitudinal and lateral reactions. In our model, we consider only the rotational and translational entropic energy. To estimate *ΔG_Entropy_*, we consider the partition function of rotational and translational motion of a cluster with N dimers,

$$Q_{N}=q_{N}^{t}q_{N}^{r};$$

where N is the total number of dimers in the cluster. The subscript $t$ stands for translation and $r$ for rotation. The entropy can then be written as

$$\begin{matrix} S_{1}=k_{B}\log Q_{1}+k_{B}T\frac{\partial}{\partial T}Q_{1} \\ S_{N}=k_{B}\log Q_{N}+k_{B}T\frac{\partial}{\partial T}Q_{N} \end{matrix}$$

where $S_{1}$ and $Q_{1}$ are the entropy and the partition function for one dimer, respectively. We approximate a dimer as a rectangular cuboid with dimensions$h_{height}\times d\times w$. A cluster has a structure (approximately) of a cuboid of dimensions$H\times D\times W$, with$H=\left\langle n_{h}\right\rangle\cdot h_{height}$, $D=d$, and$W=n_{w}\cdot w$. $\left\langle n_{h}\right\rangle$ is the average number of dimers along the longitudinal direction. $n_{w}$is the number of filaments.

Therefore, the partition function can be written as:

$$q_{N}^{t}=\left(\frac{2\pi n_{w}n_{h}mk_{B}T}{h^{2}}\right)^{3/2}V$$

$q_{N}^{r}=\pi^{1/2}\left(\frac{8\pi^{2}k_{B}T}{h^{2}}\right)^{3/2}\left(I_{hN}I_{wN}I_{dN}\right)^{1/2}$,

where *V* is the volume, and $h$is the Planck’s constant. The principal moments of inertia for a cuboid structure are

$$\begin{matrix} I_{hN}=\frac{1}{12}mn_{w}n_{h}\left(n_{w}^{2}w^{2}+d^{2}\right); \\ I_{wN}=\frac{1}{12}mn_{w}n_{h}\left(n_{h}^{2}h_{height}^{2}+d^{2}\right); \\ I_{dN}=\frac{1}{12}mn_{w}n_{h}\left(n_{w}^{2}w^{2}+n_{h}^{2}h_{height}^{2}\right). \end{matrix}$$

Combining all the equations above, we have

$$\Delta G_{Entropy}(N\to N+1)=-T\Delta S_{N,1\to N+1}=-T(S_{N+1}-S_{N}-S_{1})=F(n_{w},\left\langle n_{h}\right\rangle)\Delta G_{Entropy}(1\to2)$$

where *F*(*n_w_, n_h_*) is in general a function of *N=n_w_n_h_* (derived from the partition functions given above) that represents the entropic energy ratio of adding one dimer to the cluster with *N* dimers versus adding one dimer to another dimer. Erickson pointed out that calculating $\Delta G_{Entropy}(N\to N+1)$ directly from the corresponding partition function result in overestimation [2,3]. Instead the above relation allows us to link $\Delta G_{Entropy}(N\to N+1)$ to $\Delta G_{Entropy}(1\to2)$. The value of $\Delta G_{Entropy}(1\to2)$ is obtained by requiring that when *N* is large, $\Delta G_{Entropy}(N\to N+1)$tends to a constant value of$\sim10\text{ }k_{B}T$, as suggested by Erickson and by Howard [2,3]. The value of the overall binding free energies (~ – 9 $k_{B}T$ for longitudinal, and ~ – 5.5 $k_{B}T$ for lateral tube binding interactions) are close to what used in other model studies [4].

We want to point out that the detailed treatment of the binding energy, especially the entropic term, is not essential for the conclusion made in the main text. However, it makes the model more consistent, since the dependence of entropic change on the cluster size can affect the rates by orders of magnitude [3].

**C. Physical origins of the temperature dependence of the free energy terms:**

1) For Scheme 1, we focus on the temperature dependence of$(\Delta G_{Sh}-\Delta G_{Tu})$. Physically the potential near a stable protein conformation can be approximated as a set of harmonic potentials,

$$V=V_{0}+\frac{1}{2}\sum_{i} \kappa_{i}x_{i}^{2}$$

where $\{\kappa\}$are spring constants. The harmonic approximation makes the following analysis easy, but is unnecessary for reaching our final conclusion. The corresponding classical partition function (we neglect quantum effects which don’t change the result qualitatively here) is

$$Q=\int e^{-\frac{1}{k_{B}T}\left(V_{0}+\sum_{i} \frac{1}{2}\kappa_{i}x_{i}^{2}\right)}dx_{1}dx_{2}...dx_{N}=e^{-\frac{1}{k_{B}T}V_{0}}\prod_{i} \left(\frac{2\pi k_{B}T}{\kappa_{i}}\right)^{1/2}$$

The free energy is

$$\begin{matrix} G=-k_{B}T\ln Q \\ =-\frac{1}{2}k_{B}NT\ln T+k_{B}T\ln\left(\prod_{i} \left(\frac{\kappa_{i}}{2\pi k_{B}}\right)^{1/2}\right)+V_{0} \\ =-\alpha T\ln T+\beta(\kappa)T+V_{0} \\ \end{matrix}$$

and the free energy difference$\Delta G_{Sh}-\Delta G_{Tu}=\left(V_{S0}-V_{M0}\right)+\left(\beta_{Sh}-\beta_{Tu}\right)T$, where $\alpha$ and $\beta(\kappa)$ are positive. Therefore it is possible that$(\Delta G_{Sh}-\Delta G_{Tu})$changes sign on increasing temperature, as shown schematically in Fig. S3a.

The sign change of $(\Delta G_{Sh}-\Delta G_{Tu})$upon increasing temperature implies the entropy change$(\Delta S_{Sh}-\Delta S_{Tu}<0)$. Another possible source of entropy change is through liberation of water molecules initially bound to protein surfaces. When two protein surfaces interact, some water molecules initially constrained to the surfaces are released to the solution. This can be a huge contribution to entropy increase. Our cryo-EM images reveal more extensive contact surface for the tube bond than for the sheet bond (see Fig. 3). Therefore one might expect more water molecules released upon the tube bond formation than the sheet bond formation, which contributes the relation$(\Delta S_{Sh}-\Delta S_{Tu}<0)$. Structures at higher resolution will aid in evaluating this hypothesis. With current information, we cannot provide further quantitative analysis.

2) In Scheme 2 we assume that some conformational change (the allosteric effect) accompanies formation of two neighboring lateral bonds. Let’s denote the reaction coordinate linking the initial and final conformations *s*. The potential part of the reaction path Hamiltonian [5] along *s* can be written in the classical form

$$V(s)=V_{0}(s)+\sum_{i} \frac{1}{2}\kappa_{i}(s)x_{i}^{2}$$

The classical partition function for the potential of mean force is given by

$$Q(\bar{s})=\int e^{-\frac{1}{k_{B}T}V(s)}\delta(s-\bar{s})\prod_{i} dx_{i}ds=\exp\left(-\frac{1}{k_{B}T}V_{0}(s)\right)\prod_{i} \left(\frac{2\pi k_{B}T}{\kappa_{i}(\bar{s})}\right)^{1/2}$$

Therefore the free energy change due to the allosteric effect induced conformational change $\Delta G\equiv G(s_{b})-G(s_{0})=-k_{B}T(\ln Q_{b}-\ln Q_{0})$ is in the form$\Delta G=V_{0}(s_{b})-V_{0}(s_{0})+\left(\beta(s_{b})-\beta(s_{0})\right)T$. The term $\beta$is defined similarly to what in part 1, except here $\beta$ is dependent on conformational coordinate *s*. If$\beta_{s_{b}}<\beta_{s_{0}}$, the allosteric effect $\Delta G_{TuTu}$ decreases as temperature increase (see Fig. S3b). For simplicity we assume that only the allosteric interaction between two consecutive lateral *tube* bonds is appreciable, although the model can be easily generalized. The simulation results based on this scheme are shown in Fig. S4. The figure shows the percentage of ribbon structures at different values of$\Delta G_{TuTu}$. As discussed above, the different values of $\Delta G_{TuTu}$ correspond to different temperatures. The figure shows that smaller $\Delta G_{TuTu}$ values (higher temperature) give lower percentage of ribbon structures than larger $\Delta G_{TuTu}$ values do. For$\Delta G_{TuTu}=6k_{B}T$, the clusters contain over 90% ribbon structures, compared to the 10% for$\Delta G_{TuTu}=0k_{B}T$. The results indicate that Scheme 2 is a good alternative explanation to the existing experimental data. To discriminate between Schemes 1 and 2, more data, especially the structures with 2 PFs, would be needed.

We want to point out that entropy is the primary driving force for many biological processes, e.g., hydrophobic interactions. It is physically reasonable that the entropy term leads (*ΔG_Sh_* *− ΔG_Tu_*) to change its sign upon temperature change, especially if *ΔG_Sh_* and *ΔG_Tu_* are very close, as what we used in this work. Experimentally we found that at physiological magnesium concentration, GMPCPP tubulins form normal microtubule structure at 37^o^C, but only short single PF structures at lower temperature. These observations are consistent with our assumption that entropy has large contribution to the lateral bond energies. Increasing the temperature stablizes both types of the lateral bonds, which, esp. the sheet bond, can be further stabilized by increasing the magnesium concentration. Alternatively, one may suggest a kinetic explanation for the lacking of larger structures at lower temperature: the lateral bond formation rates are too slow. However, no larger structure is observed at longer time (in hours). This observation doesn’t support the kinetic explanation.

1. Summers K, Kirschner MW (1979) Characteristics of the polar assembly and disassembly of microtubules observed in vitro by darkfield light microscopy. J Cell Biol 83: 205-217.

2. Erickson HP (1989) Co-operativity in protein-protein association : The structure and stability of the actin filament. J Mol Biol 206: 465-474.

3. Howard J (2001) Mechanics of Motor Proteins and the Cytoskeleton. Sunderland, MA: Sinauer.

4. VanBuren V, Cassimeris L, Odde DJ (2005) Mechanochemical Model of Microtubule Structure and Self-Assembly Kinetics. Biophys J 89: 2911-2926.

5. Miller WH, Handy NC, Adams JE (1980) Reaction path Hamiltonian for polyatomic molecules. J Chem Phys 72: 99-112.

6. Wang H-W, Nogales E (2005) Nucleotide-dependent bending flexibility of tubulin regulates microtubule assembly. Nature 435: 911-915.

7. Fygensonm D, Needleman D, Sneppen K (2004) Variability-based sequence alignment identifies residues responsible for functional differences in α and β tubulin. Protein Sci 13: 25-31.

8. Sui H, Downing KH (2006) Molecular architecture of axonemal microtubule doublets revealed by cryo-electron tomography. Nature 442: 475-478.

## Supporting Figure Legends

**Figure S1. Structural basis for the two types of lateral bonds.** (a) Structure of the $\alpha\beta$-tubulin dimer with residues involved in lateral interactions indicated. Blue: residues engaged in lateral *tube* bonds (274-286, 52-61). Red: residues engaged in lateral *sheet* bond (336-342, 158-164) (these residues have been identified by docking the high-resolution tubulin structure into the 18 Å reconstruction of the ribbon [6], and therefore are correct within the constrains of the limited resolution). Pink and yellow: possible surface residues (108-130, 209-225, 300-311) along the *tube*-sheet conversion pathway. (b) Variability-based sequence alignment of $\alpha-$ and $\beta-$ tubulin performed by Fygenson et al. [7]. The blue and red boxes indicate the residues involved in the *tube* and *sheet* bond formation given in (a), respectively. The figure is adapted from Fig. 2 of Fygenson et al. [7] with permission. (c) Comparison of the non-MT lateral interactions observed in the microtubule doublet of axonemes (top) [8] (PDB file provided by Sui and Downing) and the ribbon structures (bottom) [6].

**Figure S2. Effect of variable $\Delta G_{ShSh}$ on the assembled structures with$\Delta G_{Sh}=-14.5\text{ }k_{B}T$ and $\Delta G_{Tu}=-15.5\text{ }k_{B}T$ ($\Delta G_{Sh}-\Delta G_{Tu}=1\text{ }k_{B}T>0$).** The figure shows the percentage of ribbon structures as a function of the time for $\Delta G_{ShSh}$= 0, 1, 2 and 3$k_{B}T$, as indicated.

**Figure S3. Schematic Illustration of the physical origins of the temperature dependence of the free energy terms.** (a) $\Delta G_{Sh}$and $\Delta G_{Tu}$ have different temperature dependence and their difference changes sign over *T*. (b) The dependence of $\Delta G_{TuTu}$ on the conformational coordinate describing the necessary collective conformational change upon forming two neighboring lateral *tube* bonds varies with temperature.

**Figure S4. Effects of variable $\Delta G_{TuTu}$ on the assembly structures using the Scheme 2 described in Fig. S3b.** (0, 2, 4, and 6$k_{B}T$, as indicated by corresponding circled numbers). Different $\Delta G_{TuTu}$ correspond to different temperatures as showed in Fig. S3b and supporting text C. $\Delta G_{Sh}=-13\text{ }k_{B}T$and $\Delta G_{Tu}=-16.5\text{ }k_{B}T$ were used for all simulations. Other parameters are the same as in the Scheme 1 described in detail in the main text. The final results are averaged over 60 independent simulations. (a) Percentage of ribbon structure v.s. simulation step. (b) Percentage of T-S structure. (c) Average PF length for clusters of different size (1 to 6 PFs as indicated by circled numbers), with$\Delta G_{TuTu}=2\text{ }k_{B}T$. (d) Cluster population for clusters of different size (1 to 6 PFs as indicated by circled numbers), with$\Delta G_{TuTu}=2\text{ }k_{B}T$.

**Figure S5. Population ratio of *tube*-cluster versus *sheet*-cluster for 2-PF structures as a function of time.** Solid and dashed lines with triangles correspond, respectively, to Scheme 1 ($\Delta G_{ShSh}>0$, $\Delta G_{TuTu}\sim0$, $\Delta G_{Sh}-\Delta G_{Tu}=1.5\text{ }k_{B}T$,$\Delta G_{ShSh}=6\text{ }k_{B}T$) and to Scheme 2 ($\Delta G_{TuTu}>0$, $\Delta G_{ShSh}\sim0$, $\Delta G_{Sh}-\Delta G_{Tu}=3.5\text{ }k_{B}T$,$\Delta G_{TuTu}=2\text{ }k_{B}T$), both at high temperature . The lines without triangles are for Scheme 1 (solid line,$\Delta G_{Sh}-\Delta G_{Tu}=-1.5\text{ }k_{B}T$. $\Delta G_{ShSh}=6\text{ }k_{B}T$) and Scheme 2 (dashed line, $\Delta G_{Sh}-\Delta G_{Tu}=3.5\text{ }k_{B}T$ $\Delta G_{TuTu}=6\text{ }k_{B}T$) at low temperature.
