# Supplementary figures and images for: Simulations of Tubulin Sheet Polymers as Possible Structural Intermediates in Microtubule Assembly
Source: PLoS One. 2009 Oct 2;4(10):e7291. doi: 10.1371/journal.pone.0007291 (PMC2752796; doi:10.1371/journal.pone.0007291)

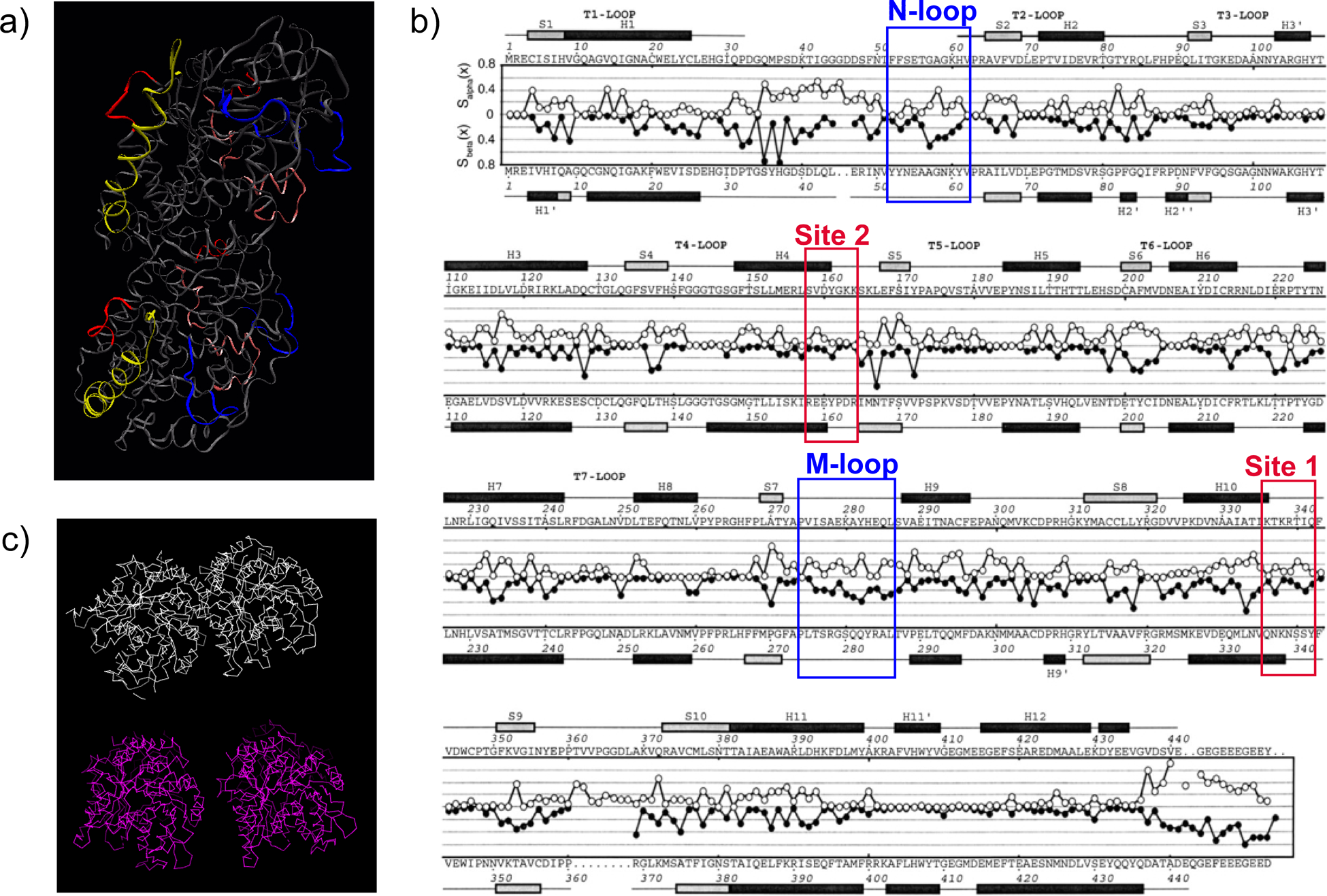

Supplement: Figure S1 — Structural basis for the two types of lateral bonds. (a) Structure of the αβ-tubulin dimer with residues involved in lateral interactions indicated. Blue: residues engaged in lateral tube bonds (274–286, 52–61). Red: residues engaged in lateral sheet bond (336–342, 158–164) (these residues have been identified by docking the high-resolution tubulin structure into the 18 Å reconstruction of the ribbon [6], and therefore are correct within the constrains of the limited resolution). Pink and yellow: possible surface residues (108–130, 209–225, 300–311) along the tube-sheet conversion pathway. (b) Variability-based sequence alignment of α and β tubulin performed by Fygenson et al. [7]. The blue and red boxes indicate the residues involved in the tube and sheet bond formation given in (a), respectively. The figure is adapted from Fig. 2 of Fygenson et al. [7] with permission. (c) Comparison of the non-MT lateral interactions observed in the microtubule doublet of axonemes (top) [8] (PDB file provided by Sui and Downing) and the ribbon structures (bottom) [6]. (1.54 MB TIF) [file pone.0007291.s002.tif]

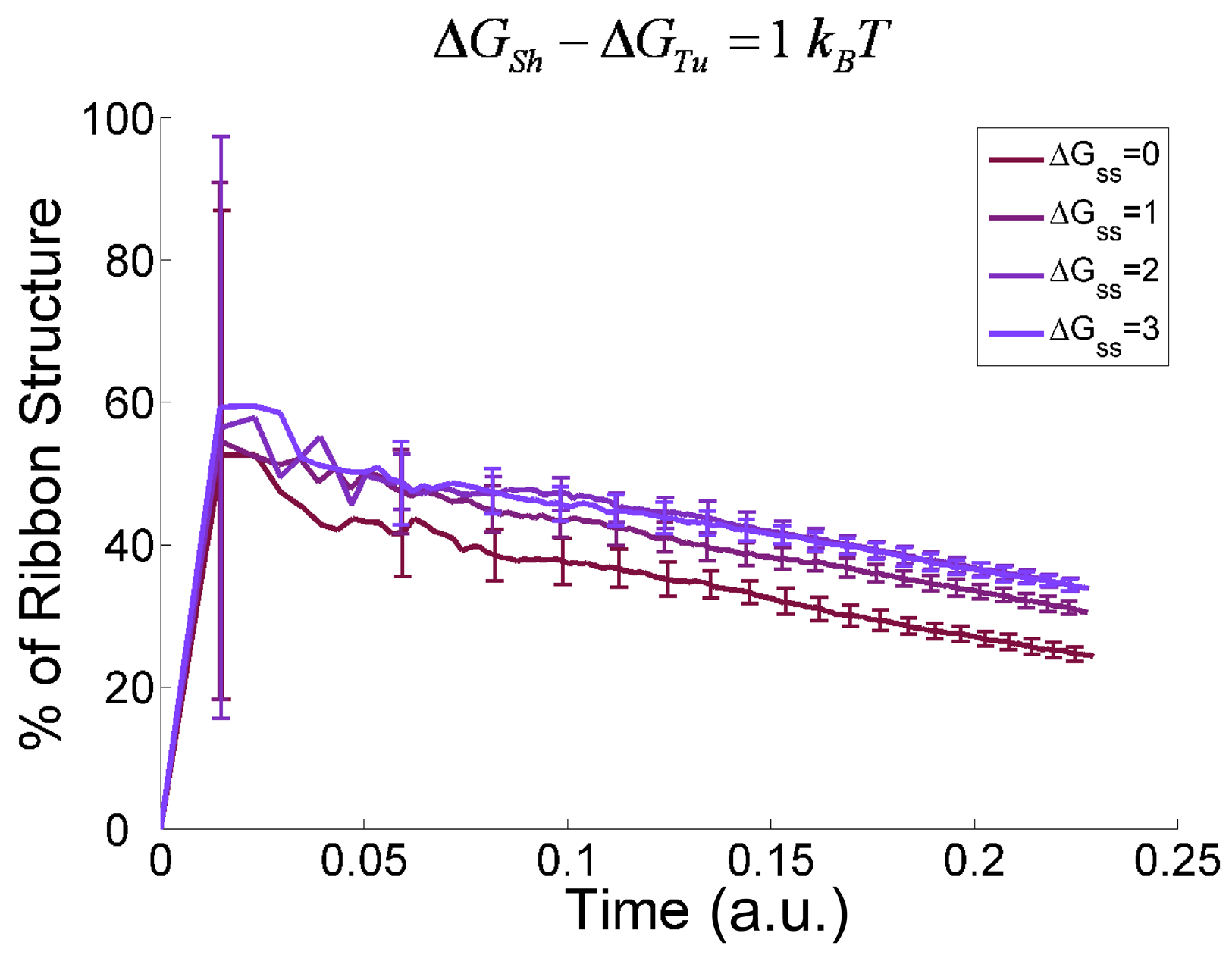

Supplement: Figure S2 — Effect of variable ΔGShSh on the assembled structures with ΔGSh = −14.5 kBT and ΔGTu = −15.5 kBT (ΔGSh−ΔGTu = 1 kBT>0). The figure shows the percentage of ribbon structures as a function of the time for ΔGShSh = 0, 1, 2 and 3 kBT, as indicated. (0.26 MB TIF) [file pone.0007291.s003.tif]

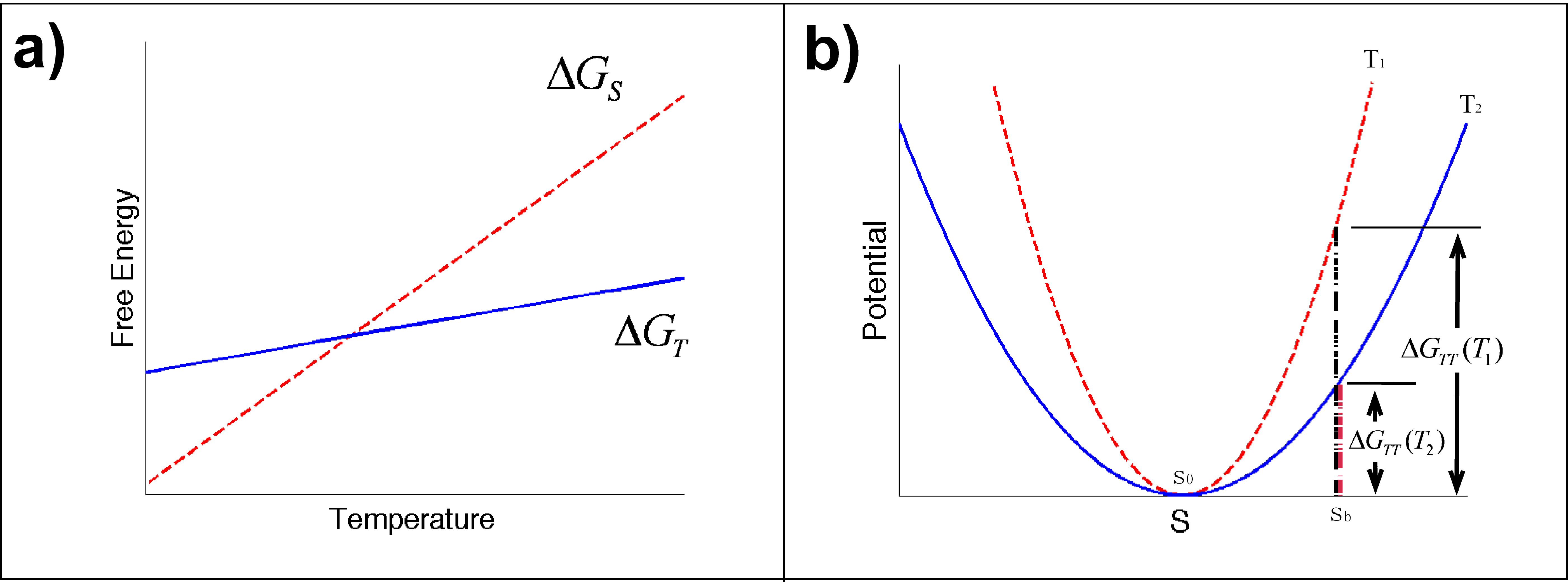

Supplement: Figure S3 — Schematic Illustration of the physical origins of the temperature dependence of the free energy terms. (a) ΔGSh and ΔGTu have different temperature dependence and their difference changes sign over T. (b) The dependence of ΔGTuTu on the conformational coordinate describing the necessary collective conformational change upon forming two neighboring lateral tube bonds varies with temperature. (0.31 MB TIF) [file pone.0007291.s004.tif]

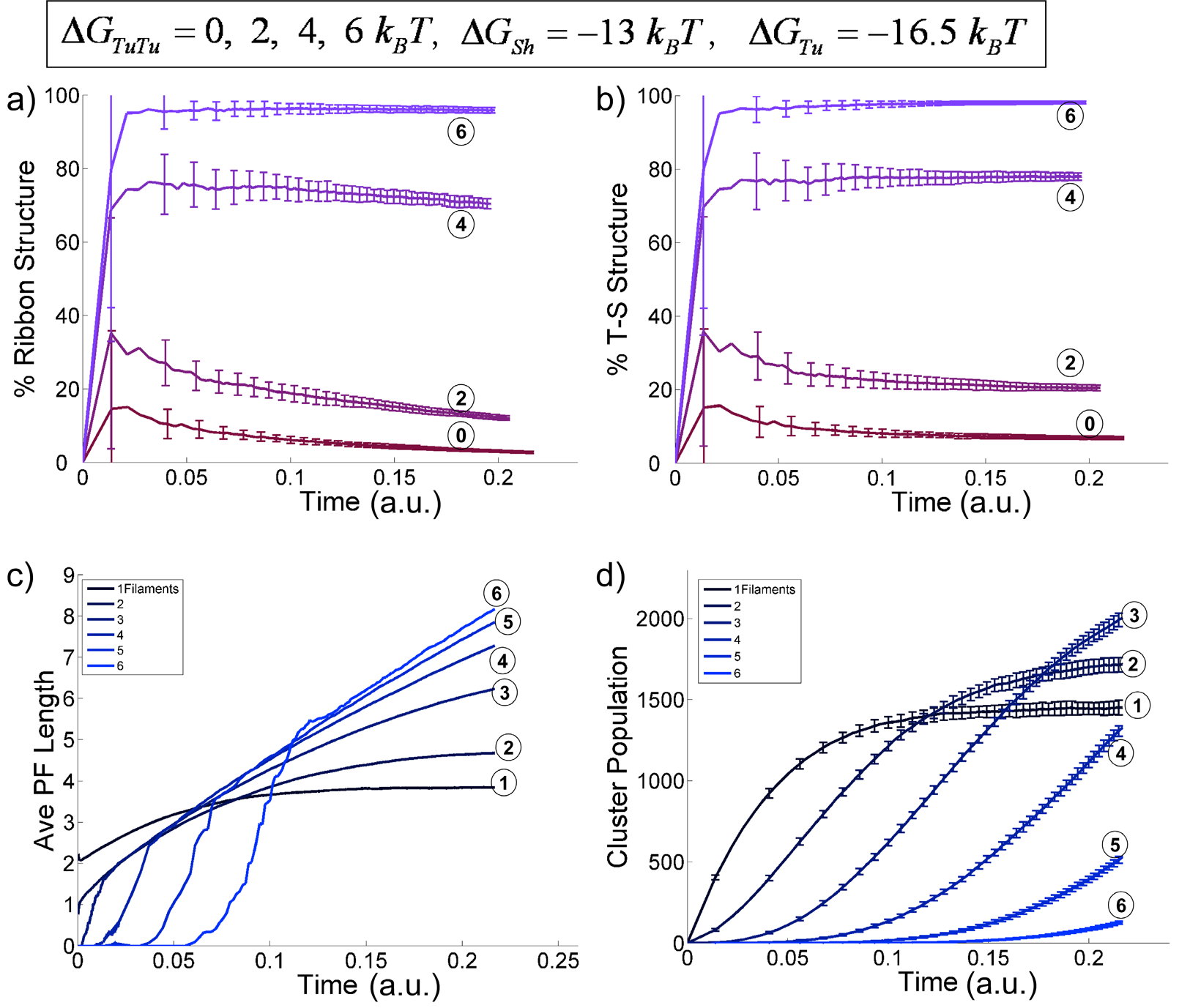

Supplement: Figure S4 — Effects of variable ΔGTuTu on the assembly structures using the Scheme 2 described in Fig. S3b. (0, 2, 4, and 6 kBT, as indicated by corresponding circled numbers). Different ΔGTuTu correspond to different temperatures as showed in Fig. S3b and supporting text C. ΔGSh = −13 kBT and ΔGTu = −16.5 kBT were used for all simulations. Other parameters are the same as in the Scheme 1 described in detail in the main text. The final results are averaged over 60 independent simulations. (a) Percentage of ribbon structure v.s. simulation step. (b) Percentage of T-S structure. (c) Average PF length for clusters of different size (1 to 6 PFs as indicated by circled numbers), with ΔGTuTu = 2 kBT. (d) Cluster population for clusters of different size (1 to 6 PFs as indicated by circled numbers), with ΔGTuTu = 2 kBT. (0.52 MB TIF) [file pone.0007291.s005.tif]

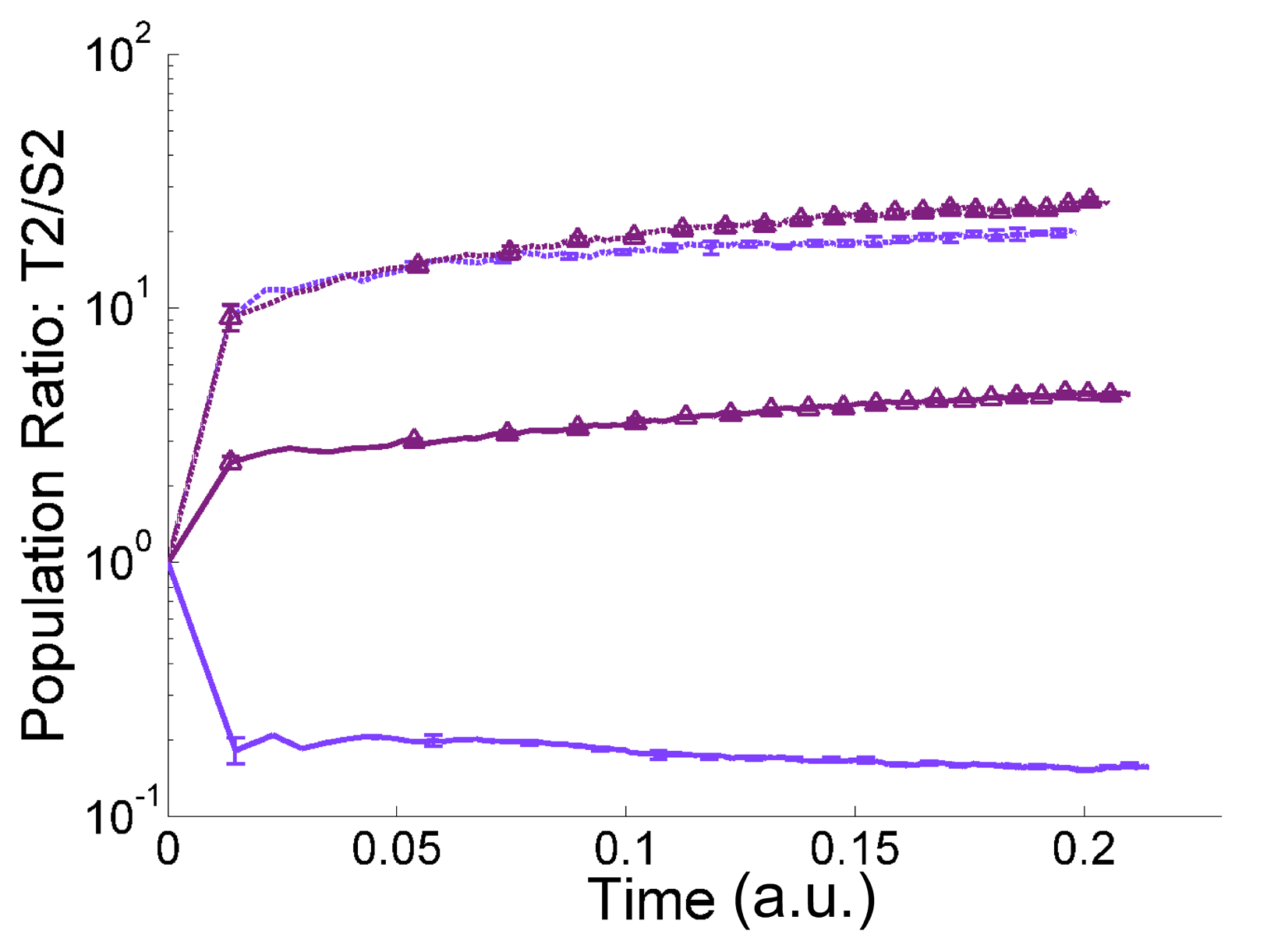

Supplement: Figure S5 — Population ratio of tube-cluster versus sheet-cluster for 2-PF structures as a function of time. Solid and dashed lines with triangles correspond, respectively, to Scheme 1 (ΔGShSh>0, ΔGTuTu∼0, ΔGSh−ΔGTu = 1.5 kBT, ΔGShSh = 6 kBT) and to Scheme 2 (ΔGTuTu>0, ΔGShSh∼0, ΔGSh−ΔGTu = 3.5 kBT, ΔGTuTu = 2 kBT), both at high temperature . The lines without triangles are for Scheme 1 (solid line, ΔGSh−ΔGTu = −1.5 kBT, ΔGShSh = 6 kBT.) and Scheme 2 (dashed line, ΔGSh−ΔGTu = 3.5 kBT, ΔGTuTu = 6 kBT) at low temperature. (0.19 MB TIF) [file pone.0007291.s006.tif]
